# Effects of Accelerated Ageing by Humidity and Heat Cycles on the Quality of Bamboo

**DOI:** 10.3390/polym14194052

**Published:** 2022-09-27

**Authors:** Hao Jia, Lei Chen, Benhua Fei, Fengbo Sun, Changhua Fang

**Affiliations:** 1 Department of Biomaterials, International Centre for Bamboo and Rattan, Beijing 100102, China; 2 Key Laboratory of NFGA/Beijing for Bamboo & Rattan Science and Technology, Beijing 100102, China; 3 Hangzhou Xinzhu Cultural & Creativity Co., Ltd., Hangzhou 310018, China

**Keywords:** humidity, heat cycle ageing, natural ageing, crystallinity, chemical properties, extension properties, thermal stability

## Abstract

The effect of humidity and heat environmental conditions on the durability of conventional bamboo materials is a pressing issue in the reserving phase of biomass materials. In this study, the relationship between the main physicochemical, pyrolytic, and mechanical properties of bamboo before and after ageing has been investigated. Exposure of engineered bamboo raw materials with moisture content up to 10% to alternating humidity and heat cycles (20 °C 98% RH-30 °C 64% RH-40 °C 30% RH) of ageing (HHT) causes degradation of the chemical polymer matrix. Byk Gardner 6840 color difference meter, X-ray diffraction, Fourier transform infrared spectroscopy (FTIR), compression intensity, thermogravimetric-infrared spectroscopy (TG-IR), and density changes are used to assess the quality of the material before and after ageing. No significant changes in the moisture content within the range of 6.12 ± 0.327 after two weeks of the engineered bamboo during wet thermal cyclic ageing were determined. However, there were significant differences in mass loss (7.75–9.93 g), cellulose crystallinity, chemical changes, compression strength, and pyrolytic properties. Differences in specimen colors were observed during 10 weeks of the accelerated humidity heat cycling ageing, and *TCD* variations ranged from 3.75 to 20.08 and from 0.25 and 3.24, respectively. Reduced cellulose crystallinity (36.459–22.638%), axial compressive strength (63.07–88.09 MPa), and modulus of rupture (2409–4286 MPa) were found during aging, whereas deformation and ductility properties were improved. Both natural and humidity heat ageing improve thermal stability and peak pyrolysis rates (0.739–0.931; 0.731–0.797). Humidity heat cyclic ageing will assist in the design and risk assessment of warehousing environments for industrial applications.

## 1. Introduction

Moso bamboo (Phyllostachys pubescens) has great economic, social, and ecological potential as an alternative to plastic and traditional materials [1,2,3]. Since bamboo is renewable, biodegradable, and has the properties to meet the basic needs of the industrial sector [4], as one of the world’s most useful natural resources, its use is growing exponentially yearly [5]. The properties of bamboo in terms of color, chemical properties, mechanical attributes, and pyrolysis characteristics are important in assessing its suitability for various end-use bamboo products. Under the same growth conditions, the annual output value of moso bamboo can reach 78.3 tons/ha (China)–4.47 times that of wood. Moreover, its sustainability is >20 times that of wood [6]. The evaluation of ageing effects of humid heat ageing environments during bamboo raw material stocking is of great interest.

Humidity and heat ageing effects usually occur with exposure to low temperature, high humidity-high temperature, and low humidity cycles. It is well-known that high-temperature environments accelerate the degradation rate of polymers [7]. The effects of high humidity on polymers include plasticization, lower glass transition temperature, increased creep and stress relaxation effects, and lower mechanical strength and elastic modulus [8,9,10,11,12,13]. The physicomechanical, chemical, and durability properties of different bamboo products have been extensively studied. Jia et al. [14] showed that the fatigue resistance of bamboo fiber asphalt mixes during accelerated ageing (0, 4, and 7 d) at 95 °C was significantly higher than that of asphalt mixes without the addition of bamboo fibers. A maximum reduction in the impact and tensile mechanical properties of bamboo/aluminum laminates by 30% could be observed for various combinations of damp and hot conditions [15]. Irina et al. [16] investigated the weathering of wood-plastic composites by damp-heat cycle, xenon arc lamp, and freeze-thaw cycle ageing. The tensile and flexural properties of the samples during ageing decreased by 2–30% compared to the specimens before ageing, whereas the addition of carbon powder to the composites slowed down the degradation rate of the products during ageing. Ma et al. [13] simulated the bending and creep properties of bamboo at a constant temperature of 20 °C for 30 d in different ambient humidity conditions. The bending and creep properties of bamboo at 90% humidity and cyclic humidity were found to be significantly higher than those of specimens at 30% humidity [17]. Although the performance of bamboo-based structural materials using accelerated ageing treatments has been evaluated in previous studies [18,19], little research has been carried out assessing the hygrothermal environmental effects on the physical and chemical properties of bamboo raw materials aging during storage.

In this study, pre-dried industrial engineered Moso bamboo samples exposed to alternating humid and heat ageing cycles in a climatic chamber were compared to those of indoor natural ageing. Accelerated ageing tests by humid heat factors were carried out, and the samples were characterized before and after these tests. The effects of ageing on materials were evaluated by colorimetry, X-ray diffraction (XRD), Fourier transform infrared spectroscopy (FTIR), compression strength, pyrolysis properties, and mass density changes.

## 2. Materials and Methods

### 2.1. Materials

The test materials were selected from 4-year-old Moso bamboo (1.5~6 m height above ground) located at the same hillside of the forest farm in Chizhou City, Anhui Province, at 120 mm diameter at breast height. A bamboo block sample of 40 mm × 20 mm × t (wall thickness) and 20 mm × 20 mm × t (wall thickness) was made as the storage specimen after removing the bamboo knots in the laboratory, which was subsequently dried in an electric heating drying kiln at 75 ± 2 °C for 30 h to obtain conventional engineering bamboo in the industry. The parameters of the single cycle program change of temperature and humidity of the storage environment were set and run alternately by the FR-1204 programmable climate chamber (Farui Instrument Technology Co., Ltd., Shanghai, China). On the other hand, indoor ageing was used as a control group specimen to simulate the natural warehousing of the engineered bamboo material.

### 2.2. Experimental Procedures

#### 2.2.1. Humidity and Heat Cycle Ageing Procedure

**Figure 1:**
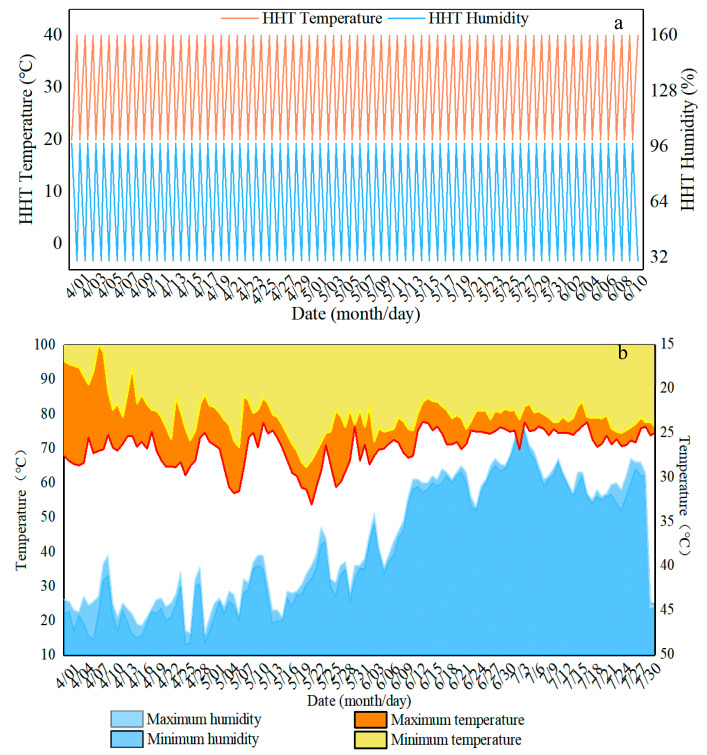
Humidity heat and natural ageing temperature and humidity monitoring. (**a**) treatment group. (**b**) Control group.

The temperature threshold of mature bamboo during indoor storage is generally 20–40 °C, with differences in the range of alternating temperature threshold intervals due to geographical differences [20]. The humidity and heat cycle ageing program adopted in this study involves the alternating change frequency of the combination of isometric temperature rise and isometric humidity decrease. The single cycle system of the climate chamber ageing program was set to 20 °C 98%RH for 8 h, followed by 30° C 64%RH for 8 h, and then 40 °C 30%RH for 8 h (Figure 1a), in which a new cycle program is automatically restarted after the last program is run. Quality trait monitoring was conducted at 1, 2, 4, 6, 8, and 10 weeks.

#### 2.2.2. Natural Ageing Procedure in the Field

Simulated natural ageing was carried indoors at 39°49’ to 40°5’ N and 116°21’ to 116°38’ E in Beijing. The temperature and humidity data were monitored online in real-time using an intelligent thermohygrometer, and the readings are shown in Figure 1b. In order to enhance the reliability of this work, samples of 4-year-old engineered Moso bamboo stored for 1 year and samples used for 3 years were also tested to assess the correlation between hygrothermal ageing and natural ageing.

### 2.3. Characterizations

The moisture content and density of the life cycle samples were tested as per GB/T15780-1995, ‘Testing methods for physical and mechanical properties of bamboos,’ for humidity heat cycle ageing and natural ageing life cycle samples. The color information, such as L* (lightness or brightness/darkness), a* (redness), or b* (yellowness), on the bamboo cortex and pith side of the sample, was quantitatively determined by BYK Gardner 6840 colorimeter. Ten sets of replicates were performed for treatment and control samples for each ageing cycle, and each replicate sample color value was measured three times, and the mean value was calculated. Their total color difference (TCD) was calculated from Equation (1).

(1) $$\mathrm{TCD}=\sqrt{{\left(L^{*}-L_{0}\right)}^{2}+{\left(a^{*}-a_{0}\right)}^{2}+{\left(b^{*}-b_{0}\right)}^{2}}$$

where L_0_, a_0_, and b_0_ indicate color values of an untreated sample.

After removing the bark and pith rings from the moisture-thermal cycle aged and naturally aged bamboo samples, the samples were made into a 60 mesh powder and analyzed using an X-ray diffractometer (X PERTPRO-30X; Philips, Amsterdam, Netherlands). The scanning angle was 5–60°, while the step size was set to 0.01° with a scan speed of 0.05°/s, and the scanning time for a single sample was 18 min 25 s. The equation for calculating the cellulose crystallinity using the peak height method is given in Equation (2).

(2) $$\mathrm{Cr}\;I=(\frac{I_{002}-I_{am}}{I_{002}})\;\times \;100\%$$

The dried bamboo powder was prepared in a Wiley mill (SHENG SHUN, high-efficiency multifunctional grinder) with a 0.25 mm sieve. FT-IR (Nexus 670 Thermo Electron Corporation) was used to monitor two categories of samples for hygrothermal cyclic ageing and natural ageing. Humidity heat ageing and the natural ageing of two sets of samples were analyzed at regular intervals on a cycle basis. Approximately 1 mg of intersegmental powder was analyzed, dried, and mixed with KBr in a ratio of 1:150 to prepare 10 mm tablets, generating spectra with a resolution of 4 cm^−1^ parameters in the range of 4000 to 400 cm^−1^ with 64 times. The FT-IR data are calibrated and smoothed by OMNIC software. The data obtained were normalized (wave number 1506) in Excel and then compared in OriginPro^®^ 2022.

Axial compression performance was tested by INSTRON mechanical testing machine with a specimen size of (20 × 20 × t) mm. In order to make the specimens fail in the range of 60–90 s, the compression loading speed was chosen as 8 mm/min. 60% of the damage was considered at the end of the test. Compression tests were performed on each group of specimens at the same loading speed, using 10 specimens for each condition, and the water content of the compressed specimens was recorded in time. Axial compressive strength was determined from Equation (3).

(3) $$F_{\left(c\right)}=\frac{P_{max}}{bt}$$

where *F*_(*c*)_ = axial ultimate compression strength, MPa; *P_max_* = maximum load, N; *b* = average width, mm; *t* = average thickness (wall thickness), mm.

The thermal stability of bamboo was assessed by thermogravimetric-infrared (TG-IR) spectroscopy. Thermogravimetric (TG) experiments were carried out using SETSYS evolutive (TGA4000, PerkinElmer, USA). A 10 mg dried intersections sample was heated at 10 °C-min^−1^ from 30 °C to 800 °C for 77 min at a nitrogen flow rate of 40 mm/min. The weight loss rates obtained were converted and normalized to the initial weight of the sample, and the data obtained were compared in OriginPro^®^ 2022.

## 3. Results

### 3.1. Color Change of Bamboo Cortex and Pith during Ageing Period

**Figure 2:**
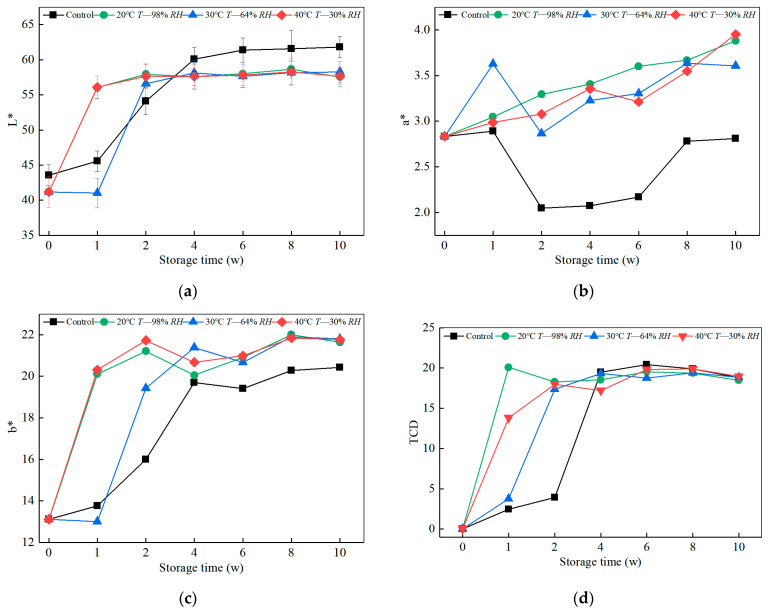
Effect of humidity and heat ageing on the color of the bamboo cortex. (**a**) Lightness values for Bamboo cortex side of all samples. (**b**) Red/green values for Bamboo cortex side of all samples. (**c**) Blue/yellow values for Bamboo cortex side of all samples. (**d**) Total color difference value for Bamboo cortex side of all samples.

In this section, an insight into the effects of climatic humidity and heat on the color of bamboo aims is provided. Visually, as the ageing cycle increases, the color changes mainly due to the lignin chromophores and co-chromophores combining in different forms with certain hydrocarbons in the bamboo and absorbing different wavelengths of visible light. It is well known that ageing methods using a combination of varying relative humidity, temperature, and treatment time can accelerate the color change of bamboo. The quantitative analysis of the surface color of cortex change (L, a, b) and the total color change (ΔE) determined by the CIELAB color space parameters are shown in Figure 2a–d, respectively. It is seen from Figure 2a that the surface brightness of bamboo green showed a tendency first to increase and then gradually stabilize with the increase of both ageing methods, reaching the maximum increase at two weeks (40.65%, 37.34%, 39.89%), six weeks (4.63%, 18.77%, 10.99%, 2.16%), followed by a variation of L* (57.96 ± 0.44) that gradually leveled off. It can be seen that humidity heat ageing plays a significant role in accelerating the brightness decay, and the brightness stabilization effect in the late ageing period (4–10 w) is better than that of the control group. The stabilization time is a full two weeks shorter than that of the control group L*. The variation in L* values (61.21 ± 0.78) stabilized after six weeks. It was greater than in the humid heat treatment group because the humid heat treatment process is a constant light-proof environment. Thus, the bamboo absorbs less UV light in this ageing environment than in the indoor control group, so the number of molecular chain breaks caused by the formation of free radicals on the surface of the bamboo with oxygen and water to form hydrogen oxides is also lower. The wavelength of UV light limits the change in color brightness. The metamorphosis rate of the surface brightness of the bamboo material is between 39.74% and 41.50% at 20–40 °C and 0–100% RH. The hygrothermal heat treatment up to 40 °C does not reduce the brightness of the green bamboo surface, which corroborates that the transition from the dark to light color of bamboo is accompanied by a gradual degradation of the chemical component lignin content.

As shown in Figure 2b, the a* values increased gradually with the hygrothermal ageing cycle by 37.02%, 28.36%, and 39.49% in each of the three ageing procedures, all of which were higher than the control group. The lower the temperature, the higher the humidity, and the higher the a* value in a single cycle. The control group showed a gradual increase in a* after the six weeks of storage, and the humidity monitoring records were above 50% RH exactly after six weeks. The relatively high humidity environment affected the redness of the bamboo green color. This ageing environment triggered an increase in surface red dimension values due to the low temperature and high humidity conditions that promoted moisture absorption on the surface of the bamboo. After eight weeks of continued ageing cycles, the a* values were no longer at a high level, which may be attributed to the hindered moisture absorption resolution, which is also reflected in the weight and density analysis section afterward.

The rate of increase in b* values during both humid heat and natural ageing tended to increase. At the beginning of ageing (two weeks), the rate of increase was higher in the humid heat treatment group (4.05, 3.16, 4.31 b*/w) than in the control group. The results show that the hygrothermal environment promotes the yellowing of the green bamboo surface. With b* values higher than 20 °C for the 30–40 °C hygrothermal program during the six-week ageing cycle, the difference between the ageing programs decreases after six weeks. High-temperature treatments greater than 150 °C significantly reduce the b* value of the bamboo surface color [21]. The outer bamboo layer is rich in vascular bundles and has fewer thin-walled cells. Thus, mild and moist heat treatment does not cause degradation of the inner layer, so the color changes occurring on the surface of the samples are usually reversible to some extent during the first six weeks of ageing.

**Figure 3:**
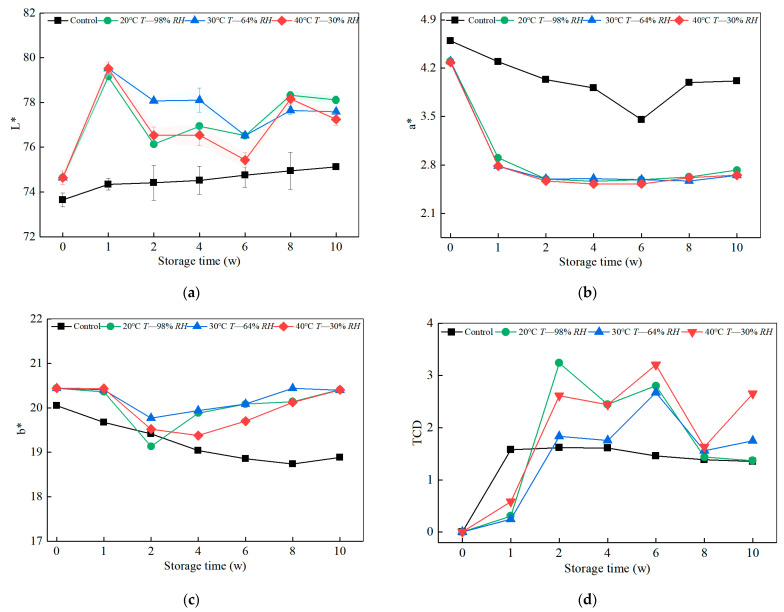
Effect of humidity and heat ageing on the color of bamboo pith. (**a**) Lightness values for Bamboo pith side of all samples. (**b**) Red/green values for Bamboo pith side of all samples. (**c**) Blue/yellow values for Bamboo pith side of all samples. (**d**) Total color difference value for Bamboo pith side of all samples.

Changes in the color of the bamboo pith under both ageing procedures were also examined. Because of the industrial applications, the bamboo pith color of the bamboo unit also affects the appearance of the final product. As can be seen from Figure 3a, L* in the indoor control group showed a gradual increase of 2.01%. The humidity and heat ageing factor had a slightly higher effect on bamboo yellow L* values than indoor UV, averaging 3.36% higher than the control after 10 weeks of ageing cycles. The effect of humidity and heat ageing on the a* values of bamboo yellows showed a decreasing and leveling trend. The decrease was higher than the control group, and the differences between the humidity and heat ageing procedures were small (Figure 3b). The bamboo pith side has a higher proportion of thin-walled cell tissue than fibrous tissue. The contribution of light ageing factors due to structural differences in gradients is lower than that of hygrothermal ageing factors. The humidity heat ageing factor has a greater effect on the bamboo yellow b* values than the indoor light ageing factor. The color difference values show that the effect of the indoor visible light ageing factor on the differential bamboo yellow values is determined within one week of storage. However, the damp heat ageing factor has a cyclical and alternating effect, and is greater than the control group for the same period, with a correlation with moisture absorption and dissipation at a certain ageing stage.

### 3.2. Effect of Humid Heat Cycle on the Weight and Density of Bamboo during Accelerated Ageing

**Figure 4:**
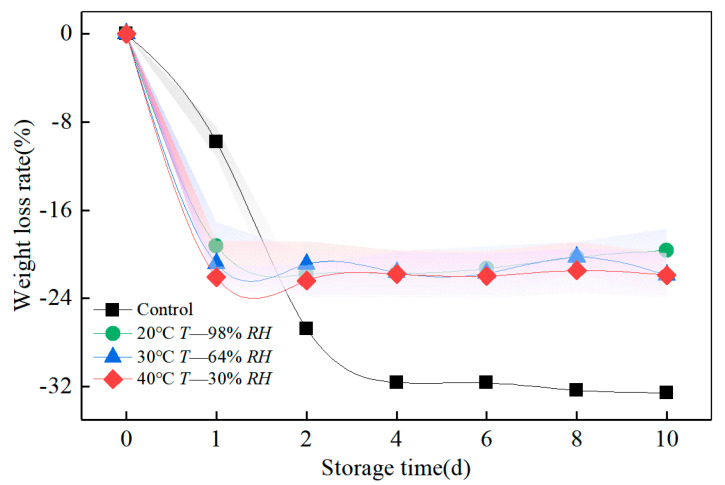
Effect of humidity and heat ageing on the weight of bamboo.

Table 1 and Figure 4 show the variations in the weight of the bamboo blocks during 10 weeks of humidity, heat ageing, and indoor storage. The trend of the weight loss rate during the humidity heat cycle ageing procedure is in the order of 40 °C, 30% RH > 30 °C, 64% RH > 20 °C, 98% RH. At the beginning of ageing, the reduction of internal surface voids inhibits the entry of water molecules as the bamboo blocks are dried in the kiln. Moreover, the hygroscopic capacity of the blocks is reduced, which gradually increases after the sixth week. The environment during storage in the indoor control group was ventilated and relatively constant. The humidity during the first four weeks of the storage cycle was below 30% RH, and the rate of moisture loss was faster. Still, the weight remained stable as the ventilated environment was not conducive to moisture absorption by the bamboo.

**Figure 5:**
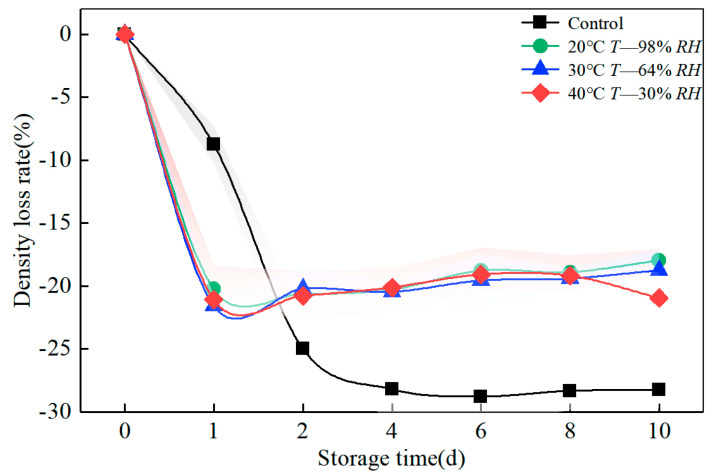
Effect of humidity and heat ageing on the density of bamboo.

The change in density (Figure 5 and Table 2) corresponds to the weight, and it is worth noting that the density is slightly higher for the wet-heat cycle procedure at 40 °C, 30% RH, than for the other two procedures. The reason for this attribute may be that, in addition to moisture dissipation, dimensional expansion affects changes in density. When the bamboo block absorbs moisture at 98% RH at 20 °C followed by moisture dissipation at 30% RH at 40 °C may cause dimensional expansion of the block, it seems that after six weeks, the dry shrinkage and wet swelling phenomenon gradually declines (9.65%). As a hygroscopic and fiber-based plant material, the properties of bamboo blocks significantly impact the durability, indoor hygiene, and energy consumption of buildings. Many researchers have estimated the fiber saturation point of bamboo at 13–20% [22], and moisture content during growth (80%) [23] and moisture transfer and opening rates of different species of bamboo [24] are also discussed in detail. However, most current studies are based on fresh bamboo as a test material, and there are still differences in the practical application of industrial bamboo. When bamboo is cut and stored dry, its moisture content is lower than that of wood. The water storage capacity and moisture transport rate of bamboo are generally lower than that of an equivalent volume of wood product. This research confirms that the wet shrinkage and swelling effect of engineered bamboo below 10% after drying at 20–40 °C 30–98% RH gradually increases after a six-week storage period. 

### 3.3. Effect on Cellulose Crystallinity during Humid Heat Cycle Ageing of Bamboo

**Figure 6:**
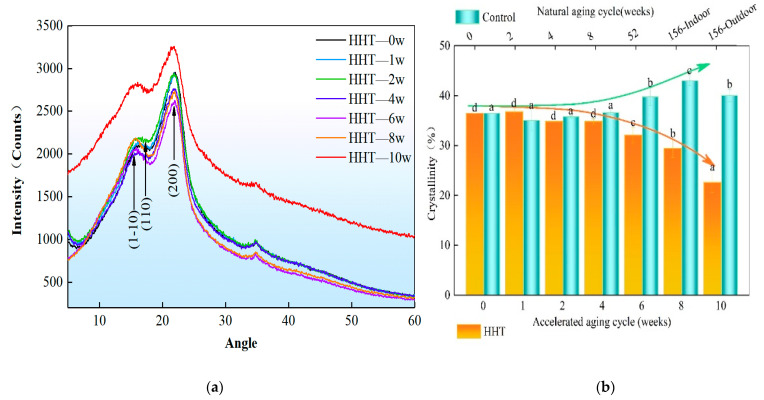
XRD patterns of bamboo during humid and heat cycle ageing. (**a**) Diffraction of x-rays patterns of all samples. (**b**) Cellulose crystallinity of all samples measured by peak height method. Note: Different lowercase letters between the two groups indicate significant differences (*p* < 0.05).

The hygroscopic resolution cycle of bamboo impacts the cellulose crystallinity, which reflects the physical properties of the fiber to some extent. In cellulose microfilaments with crystalline and non-crystalline (amorphous) zones, the percentage of the crystalline zone in the overall cellulose microfilaments is called the crystallinity of the bamboo material. In general, the moisture absorption capacity of bamboo is negatively correlated with crystallinity [25]. The bamboo was subjected to a pre-set ageing program with a damp heat cycle, and the samples were air dried at 25 °C-46% RH at each monitoring cycle for crystallinity testing. From Figure 6a, it can be observed that the α-cellulose of bamboo contain mainly two peaks. The first one is a multiple and width of 2 = 14.88 and 2 = 16.09, referring to the (1–10) and (110) crystal planes, respectively; The second peak is sharp and strong at 2 = 22.86. It shows that as the damp heat ageing cycle increases, the peak signal intensity at angle 2θ tends to decrease and then increase, dropping to a minimum at cycle 42d. The cellulose crystallinity, calculated by the peak height method, showed a gradual decrease, with the ANOVA (Table 3 and Table 4) showing a significant decrease after the fourth week, in the order of 7.89%, 8.36%, and 23.02% (Figure 6b). This corresponds to the weight change rates mentioned earlier, where the moisture absorption capacity of bamboo blocks subjected to cyclic ageing procedures at low temperature and high humidity (20 °C and 98% RH) and high temperature and low humidity (40 °C and 30% RH) was treated in a steady state during the first four weeks. It was also stimulated after the sixth week, becoming rapid in moisture dissipation under high temperature and low humidity procedures and strong in moisture absorption under low temperature and high humidity. It also validates the decrease in wetness with an increase because the crystalline zone does not absorb water. The ability of the non-crystalline zone to absorb water is inversely proportional to the degree of crystallinity, which is consistent with the findings of Stokke et al. (2013) [26] and Rowell et al. [27]. Meanwhile, the control specimens monitored for natural indoor ageing were not significantly different during the eight-week storage period, with stable control of cellulose crystallinity of (35.97 ± 0.72) % and increases of 10.58% 19.57% after one and three years of indoor storage, respectively. During indoor storage, direct sunlight and rainwater are avoided, so the crystallinity caused by moisture and oxidation in the air-dried state increases and the ability to absorb moisture becomes weaker. The orderly deposition of cellulose and lignin during growth is the main factor influencing the crystallinity. The crystallinity of cellulose is influenced by the differentiation and degradation of lignin in the crystalline or non-crystalline zones during storage. Samples placed outdoors for three years are less crystalline than those stored indoors for three years, and bamboo stored for three years is more weathered in the outdoor environment than in the indoor environment. The high lignin content of bamboo helps it to maintain its structural integrity during storage [28], e.g., indoor storage. In conclusion, the short-term hygrothermal ageing for one month is inadequate in affecting the crystallinity of bamboo cellulose.

### 3.4. Effect of Bamboo on FT-IR during Humid Heat Cycling and Natural Indoor Ageing

**Figure 7:**
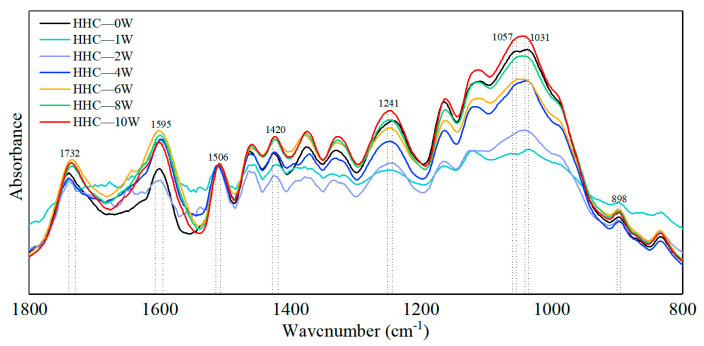
Fourier infrared spectra of bamboo during humidity and heat ageing.

As the main components in the engineered bamboo samples are cellulose, hemicellulose, and lignin, the spectra of all three are shown in Figure 5, together with the spectra of the bamboo powder. The wavenumber ranges where the characteristic peaks of the three major functional groups are located are shown in Table 5. All samples were dried before testing and normalized at the 1506 wave number position where the signal was most pronounced, and the plots were meaningfully comparable throughout the monitoring cycle. Macroscopically, accelerated ageing appears to alter the chemical composition of the bamboo samples slightly. Lignin is known for its aromatic and hydrophobic properties. It is composed of three phenyl propane units (S-, G-, and H-units) in a complex, stable and diverse structure of amorphous three-dimensional bulk macromolecules. The absorbance at wave numbers (1033, 1241, 1420), where the main component is the lignin characteristic functional group, showed a sharp decrease followed by a gradual increase. The partial stretching pairs (1595) in the aromatic lignin skeleton were all enhanced during hygrothermal ageing, echoing Figure 7, with the phenolics and phenolic syringyl groups in lignin first decreasing from 32.34% and 22.05% before ageing to a minimum of 21.75% and 17.98, respectively, before gradually rebounding to 30.64% and 21.18%. The aromatic functional groups showed a fluctuating upward trend during the hygrothermal ageing process and were all 16.85% higher than the pre-ageing specimens. In addition, the characteristic stretching pairs of the benzene ring aromatic skeleton and the aromatic part increased and then decreased. The rapid water dissipation in bamboo blocks with a moisture content of 10% may be accompanied by an unstable expression or reversible destruction of the molecular formula of the aromatic skeleton when induced by humidity and heat ageing factors so that the hygrothermal ageing effect starts in an activated state and gradually decreases with increasing ageing time. The increased lignin absorption peak of the aged biomass material is attributed to the oxidative modification of lignin by reactive oxygen species derived from the Fenton reaction [29]. Humid heat treatment causes gradual degradation of xylan in bamboo hemicellulose [30]. Poletto et al. [31] demonstrated that heat treatment breaks the β-O-4 structure in lignin, leading to an increase in etheric and phenolic structures. The increase in phenolic structure is probably the main factor in the increase of the absorption peak of the characteristic functional groups in the later stages. However, this ageing temperature is much less than the irreversible degradation temperature of bamboo (200 °C) [32], and it is a small change in the functional group of a chemical substance.

**Figure 8:**
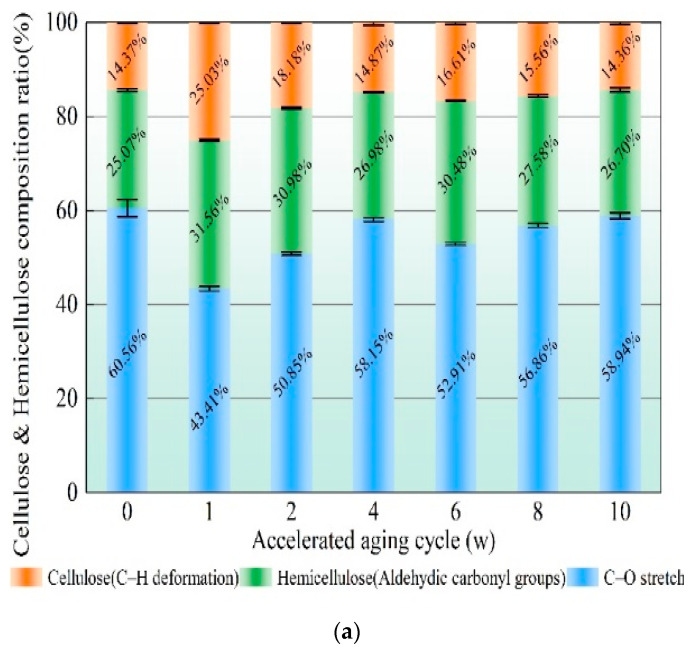

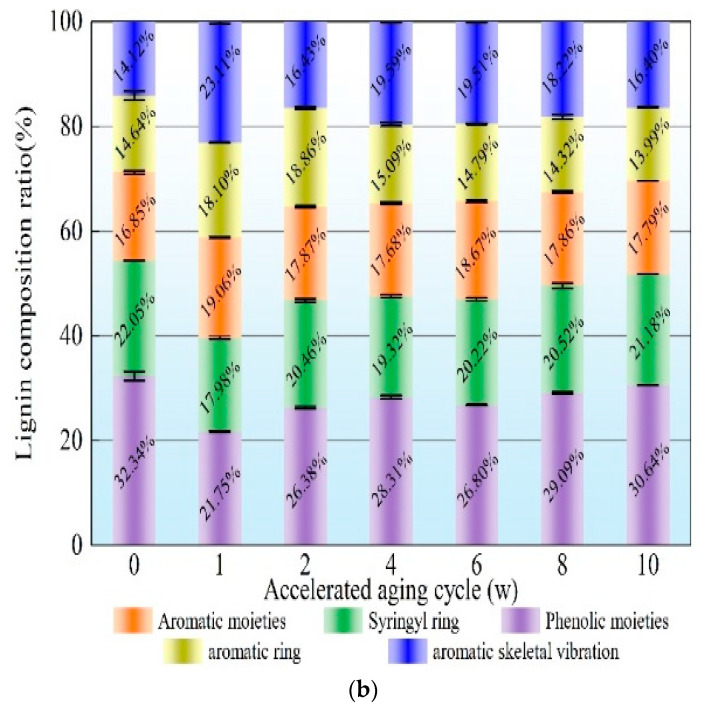
Fourier infrared spectra of bamboo during humidity and heat ageing. (**a**) Chemical composition of cellulose and hemicellulose functional groups as a percentage (**b**) Chemical composition of lignin functional groups as a percentage.

The absorption peaks of cellulose C-H deformation during humidity heat ageing show a decreasing trend followed by an increasing trend. The trend of the overall composition percentage is shown in Figure 8a,b. The absorption peak for the one-week ageing cycle is at a higher rate of moisture loss than absorption under the humidity heat ageing procedure, as confirmed in the previous section on weight density change. The C-H deformation is affected by a sharp decrease in moisture when moisture is dissipated from the interior of the bamboo mass and the intensity of the absorption peak of characteristic functional groups such as cellulose increases. The C-H deformation is affected by a sharp decrease in moisture when moisture is dissipated from the interior of the bamboo mass and the intensity of the absorption peak of characteristic functional groups such as cellulose increases.

**Figure 9:**
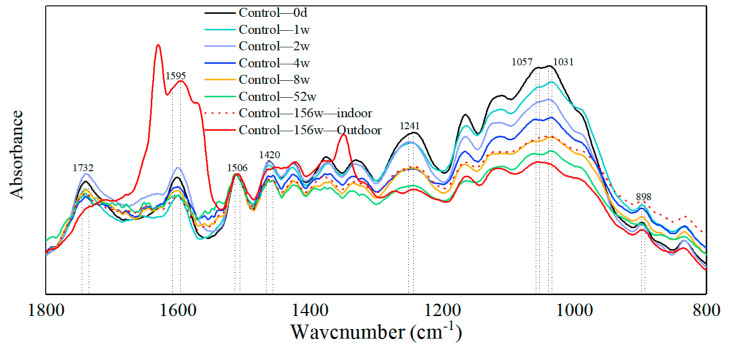
Fourier infrared spectra of bamboo during natural indoor ageing.

In this study, additional Fourier spectra of chemical composition were monitored during natural ageing (Figure 9). With the extension of the ageing cycle, the lignin functional group tends to decline gradually. In the natural ageing storage period, the room temperature is constant, the moisture loss is also gentle, and the lignin functional group gradually declines during oxidative degradation. However, the degradation of the samples stored outdoors was greater than those stored indoors. It is noteworthy that the stretching pair absorption peak near the characteristic aromatic functional group of the samples stored outdoors for three years was significantly higher than that of the other ageing groups, indicating the possibility of aromatic compounds being produced in the samples stored outdoors. Unlike accelerated humidity and heat ageing, the absorption peaks of the C-O stretch and C-H deformation characteristic functional groups of hemicellulose and cellulose decreased gradually during natural ageing. Hemicellulose xylan showed a gradual decrease overall and the lowest trend for samples stored outdoors for three years. Therefore, long-term storage of bamboo in its original state is recommended to be carried out indoors, away from decaying fungal substances and direct contact with rain and UV light. In this way, the degradation of hemicellulose and the production of lignin aromatic functional groups during the stocking of engineered bamboo will be avoided as far as possible.

### 3.5. Effect of Bamboo on Axial Compressive Properties during Humid Heat Cycling and Natural Indoor Ageing

**Figure 10:**
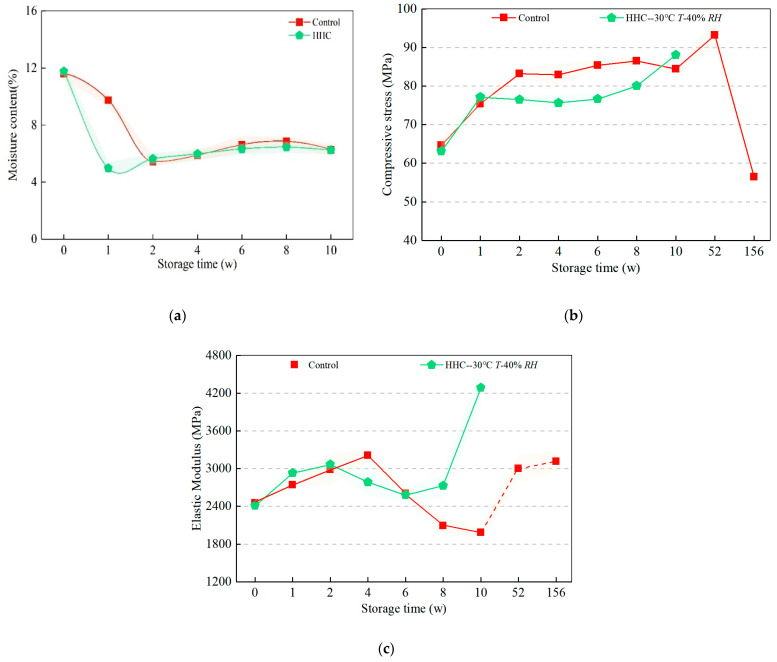
Effect of humidity heat cyclic ageing on moisture content and axial compression properties of bamboo. (**a**) Moisture content of the treated and control group samples during storage. (**b**) Compressive stress of treated and control group samples during storage. (**c**) Elastic modulus of treated and control group samples during storage.

The mechanical properties of bamboo are closely related to its chemical properties. The mechanical strength of bamboo decreases with increasing moisture content. The moisture content of the material was maintained at 6.12% after two weeks in both ageing methods (Figure 10a). The axial compressive strength of the samples is increased at the beginning of ageing due to the dissipation of the moisture content. However, the reduction of the composition of the characteristic functional groups, such as lignin of the bamboo under the hygrothermal ageing cycle mentioned earlier, indirectly increases the proportion of hemicellulose and cellulose of the three major elements in the material, and thus the compression strength. The contribution of cellulose to axial compression strength is known to be large [36]. The compressive strengths obtained from the two ageing methods are distinguishable after one week and gradually increase in a parabolic pattern over the ageing cycle of 2–8 weeks, with a 2.47% decrease in the compressive strength of the control group and a 4.38% increase in the damp heat cycle treated group after 10 weeks (Figure 10b). The signal intensities of the three major characteristic functional groups of the samples at the end of the hygrothermal ageing are at a high level throughout the ageing phase. As can be seen in the elastic modulus graph (Figure 10c), both forms of ageing appear to have the same pattern of change, with both showing a trend of increasing, then decreasing, and then increasing again. However, hygrothermal ageing accelerates the time at which the modulus of elasticity of the bamboo block reaches its first peak and trough, which may be valuable for predicting future trends in the modulus of bamboo during the one-year reserve period. After three years of storage, the compression properties of the bamboo material drop dramatically by 42.60% compared to bamboo stored for one year, which tells us that the storage period for engineered bamboo without any protective treatment should not exceed three years. Regular care and storage are essential.

**Figure 11:**
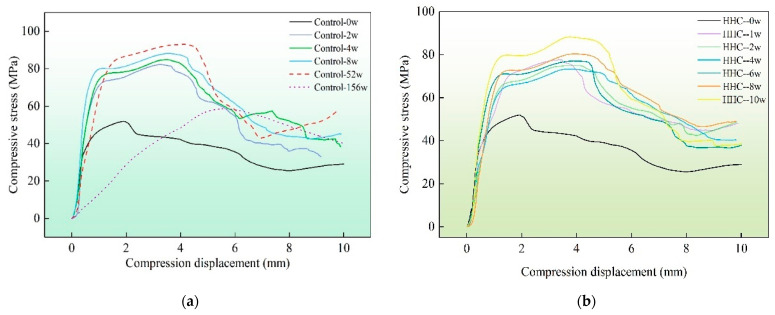
Stress-strain curve during humidity heat ageing and natural ageing. (**a**) Stress-strain curves during storage of control group samples. (**b**) Stress-strain curves during storage of treatment group samples.

In order to present further details of the specimen during compression, typical stress-strain diagrams during ageing were extracted. As can be seen in Figure 11a, as the natural ageing time (52 weeks) increases, the slope of the compression curve gradually increases the maximum compression strength from 51.83 MPa to 92.81 MPa, and the displacement to the peak increases gradually (1.93, 3.31, 3.56, 3.61, 4.29 mm). On the other hand, the peak of the stress–strain curve during hygrothermal ageing tends to decrease and then increase, with a maximum decrease of 9.82% (four weeks), followed by increases of 3.68%, 5.52%, and 9.51%, and a gradual increase in the maximum increase and the displacement to the peak stress (3.33, 3.60, 3.61, 3.84, 3.93, 3.94 mm). This indicates that the bamboo ductility gradually increases during hygrothermal ageing and indoor storage, and the hygrothermal ageing factor is more likely to stimulate the ductility of engineered bamboo in the same ageing time frame. The change in ductility is used as a major criterion for the ageing performance of bamboo blocks. Ageing conditions of alternating humidity and heat cycles should be avoided during stocking.

### 3.6. Effect of Bamboo on Axial Compression Properties during Humid Heat Cycling and Natural Indoor Ageing

**Figure 12:**
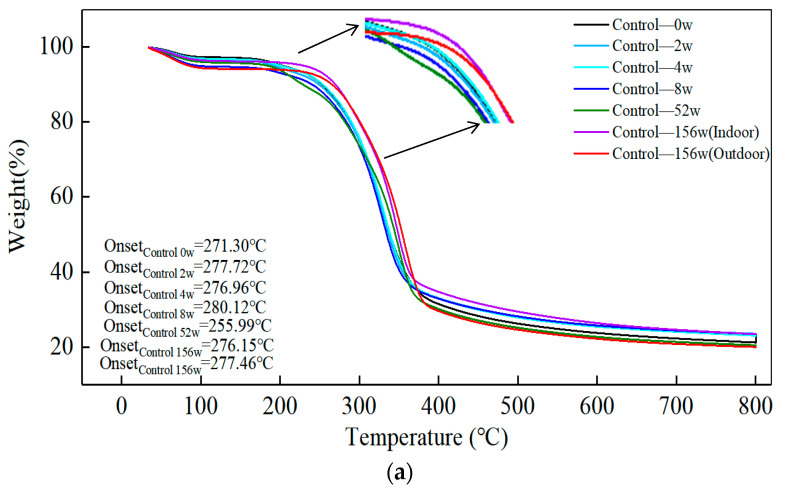

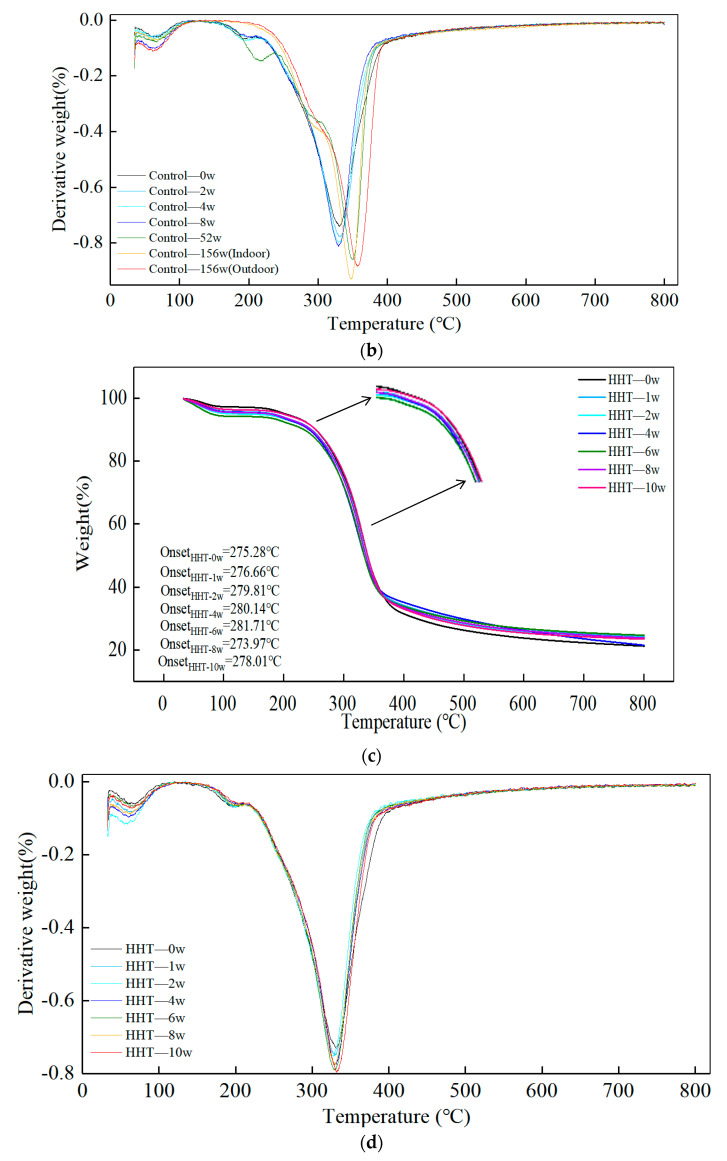
Effect of humid heat ageing and natural ageing on the thermal stability properties of bamboo. (**a**) Thermogravimetric analysis of control group samples during storage (**b**) Derivative Thermogravimetry of the control group samples during storage. (**c**) Thermogravimetric analysis of treatment group samples during storage. (**d**) Derivative Thermogravimetry of the treatment group samples during storage.

Pyrolysis of biomass materials has become an emerging and popular research area. There are many research studies conducted on the biomass pyrolysis processes. However, little focus has been put on the pyrolytic properties during warehouse storage, where thermal stability is also an important indicator for assessing bamboo during storage. The pyrolysis process during hygrothermal ageing (10 weeks) and natural ageing (156 weeks) was analyzed by TGA and DTG curves. The curve (Figure 12a,c) illustrates the three stages of degradation. In the first stage (25–150 °C), weight loss occurs due to water evaporation. In the second stage (250–300 °C), pyrolysis of hemicellulose mainly occurs. In the third stage (300–350 °C), pyrolysis of cellulose mainly occurs [37]. This section focuses on the hygrothermal ageing effect and the pyrolytic properties of bamboo under natural indoor ageing.

As the ageing time increases, the peak pyrolysis temperature of bamboo stored indoors does not change significantly for eight weeks and is around 330.30 °C. The initial pyrolysis temperature, however, tends to increase slightly in sequence. The peak pyrolysis temperatures for 52–156 weeks increased significantly from 349.49 °C, 348.50 °C, and 357.10 °C, respectively. The peak pyrolysis rates increased sequentially to −0.739, −0.776, −0.797, −0.812, −0.859, −0.931, and −0.882, respectively. This confirms the findings presented in the preceding sections, as shown by the cellulose characteristic functional group. The signal intensity gradually decreases, corroborating the decrease in cellulose characteristic functional groups. The maximum pyrolysis rate is influenced by the decrease in cellulose content. The maximum pyrolysis rate increases, and the thermal stability gradually decreases. The intensity of the signal of lignin and cellulose characteristic functional groups of bamboo placed indoors positively correlates with the pyrolysis rate and temperature. Bamboo stored outdoors is less likely to maintain its original pyrolysis temperature and, in practice, reaches a higher exothermic temperature than raw materials stored indoors.

The initial pyrolysis temperature of the bamboo under moist heat ageing showed an increasing trend followed by a decreasing trend. Thermal stability reached its optimum at the 6th week of ageing, and the peak pyrolysis rate showed a gradual increase with −0.731, −0.749, −0.753, −0.775, −0.776, −0.790, −0.797. Humidity heat cycle ageing theoretically enhances the exothermic efficiency of the biomass fuel (bamboo) due to peak pyrolysis temperature fluctuations in the range of (329.89 °C ± 1.56). The bamboo stock can be stored in alternating cycles of low temperature and high humidity and high temperature and low humidity, which improves its thermal stability. When bamboo is utilized in a graded classification, the bamboo used for biomass fuel has a broader threshold of resistance to humid thermal environments.

## 4. Conclusions

In summary, the environmental stability of engineered bamboo materials after drying in industrial kilns was evaluated using hygrothermal cyclic ageing and natural ageing. The results showed that the hygroscopic resolution of the bamboo blocks gradually stabilized during the first four weeks of the ageing cycle. With higher a* and b* values and lower L* values than the natural indoor ageing at 20 °C-98%RH-8 h, 30 °C-64%RH-8 h, and 40 °C-30%RH-8 h. The total color difference values and modulus of elasticity of the hygroscopically aged samples changed situation two weeks earlier than the control group. After six weeks, the ageing fatigue forces the moisture evaporation and transport between the inside of the bamboo block and the outside due to humidity and heat. Moreover, the cellulose crystallinity tended to decrease gradually during hygrothermal ageing (7.89–23.02%). Lignin characteristic functional groups tended to decrease and then increase, whereas the aromatic characteristic functional groups increased in signal intensity. The cellulose characteristic functional groups increased in absorption peak intensity. The cis-grain compression strength decreased by 22.28–39.68%. The peak pyrolysis rate was 5.01–25.98%. During natural ageing, the samples showed an increasing trend in cellulose crystallinity (10.58–19.57%), varying degrees of reduction in the characteristic functional groups of lignin and hemicellulose, a 12.64% reduction in paracellular compression strength, and a reduction in the peak pyrolysis rate in the range of 5.01–19.35%.

## Figures and Tables

**Table 1:** Humid heat cycle ageing data records on bamboo weight response.

	Treatment	Dimensions (L × W × H)/mm	30 °C *T*-64% RH/g	40 °C *T*-30% RH/g	20 °C *T*-98% RH/g	Control/g
Ageing Cycle (Weeks)						
0		39.65 × 21.45 × 13.60	9.93 ± 0.277	9.93 ± 0.277	9.93 ± 0.277	8.92 ± 0.422
1		39.56 × 21.51 × 13.57	7.86 ± 0.269	7.82 ± 0.267	8.02 ± 0.264	6.52 ± 0.279
2		39.44 × 21.03 × 14.59	7.85 ± 0.263	7.75 ± 0.259	7.76 ± 0.256	6.24 ± 0.233
4		39.31 × 22.77 × 13.58	7.78 ± 0.256	7.76 ± 0.257	7.77 ± 0.257	6.15 ± 0.224
6		38.96 × 20.81 × 13.42	7.77 ± 0.256	7.75 ± 0.256	7.81 ± 0.259	6.18 ± 0.158
8		38.89 × 20.92 × 13.42	7.91 ± 0.450	7.91 ± 0.480	7.75 ± 0.256	6.20 ± 0.219
10		38.76 × 20.92 × 13.57	7.75 ± 0.256	7.98 ± 0.258	7.75 ± 0.256	6.18 ± 0.217

**Table 2:** Humid heat cycle ageing data records on bamboo density response.

	Treatment	Weight/g	Volume/cm^2^	30 °C *T*-64% RH	40 °C *T*-30% RH	20 °C *T*-98% RH	Control
Ageing Cycle (Weeks)							
0		9.626	11.717	0.848 ± 0.022	0.847 ± 0.022	0.847 ± 0.022	0.914 ± 0.025
1		7.855	11.747	0.669 ± 0.014	0.668 ± 0.015	0.675 ± 0.012	0.885 ± 0.015
2		7.852	11.621	0.676 ± 0.013	0.671 ± 0.012	0.672 ± 0.013	0.745 ± 0.233
4		7.778	11.473	0.678 ± 0.014	0.677 ± 0.014	0.675 ± 0.013	0.708 ± 0.020
6		7.768	11.305	0.687 ± 0.015	0.700 ± 0.021	0.688 ± 0.012	0.704 ± 0.018
8		7.912	11.388	0.694 ± 0.019	7.91 ± 0.480	7.75 ± 0.256	0.708 ± 0.018
10		7.753	11.381	0.681 ± 0.015	7.98 ± 0.258	7.75 ± 0.256	0.705 ± 0.012

**Table 3:** Values of cellulose crystallinity during aging of bamboo ANOVA.

Group Category			Accelerated Aging Treatment Group					Natural Aging Control Group				
			Quadratic Sum	df	Mean Square	F	Signficance	Quadratic Sum	df	Mean Square	F	Signficance
Inter-group	Combination		457.021	6	76.17	50.446	0	151.119	6	25.186	17.247	0
	Linear term	contrast	373.557	1	373.557	247.401	0	100.477	1	100.477	68.805	0
		deviation	83.464	5	16.693	11.055	0	50.642	5	10.128	6.936	0.002
Intra-group			21.139	14	1.51			20.444	14	1.46		
Total			478.16	20				171.563	20			

**Table 4:** Numerical significance analysis of cellulose crystallinity during aging of bamboo.

Accelerated Aging Treatment Group							Natural Aging Control Group					
	Sample Number	Number of Cases	Subset of Alpha = 0.05					Sample Number	Number of Cases	Subset of Alpha = 0.05		
	Aging Period (Weeks)		1	2	3	4		Aging Period (Weeks)		1	2	3
Duncan’ a	10	3	22.6381				Duncan’ a	2	3	35.0346		
	8	3		29.409				3	3	35.761		
	6	3			32.0932			1	3	36.4589		
	4	3				34.8435		4	3	36.6047		
	2	3				34.8734		5	3		39.7758	
	0	3				36.4589		7	3		40.0109	
	1	3				36.8112		6	3			43.0109
	Significance		1	1	1	0.091		Significance		0.163	0.815	1

**Table 5:** Assignment of significant functionalities from FTIR spectra.

Wavcnumber (cm^−1^)	Vibration	Funcalities	Component	References
898	C–H deformation	—	cellulose	[28,33]
1031	C-O stretching	Phenolic moieties	Lignin	[34]
1057	C–O stretch	—	Cellulose and hemicellulose	[35]
1241	C–O–C stretching	Syringyl ring	Lignin	[33]
1420	–(Ar) C=C stretching	Aromatic moieties	Lignin	[34]
1506	aromatic skeletal vibration	Aromatic ring	Lignin	[35]
1595	–(Ar)C=C	Aromatic skeletal vibration	Lignin	[33]
1732	–(H)C=O stretching	Aldehydic carbonyl groups	Hemicellulose (xylan)	[35]

## Data Availability

The data presented in this study are available on request from the corresponding author.
